# Stenotrophomonas maltophilia in Hemodialysis: An Opportunistic Pathogen or a Malevolent Foe

**DOI:** 10.7759/cureus.73277

**Published:** 2024-11-08

**Authors:** Tamilarasan V, Jagan V, Leela KV, Arunkumar Asokan, Mathew Gerry George, Varadharajan Jayaprakash

**Affiliations:** 1 Microbiology, SRM Medical College Hospital and Research Centre, Chennai, IND; 2 Nephrology, SRM Medical College Hospital and Research Centre, Chennai, IND

**Keywords:** catheter-related bloodstream infection (crbsi), colonic ulcers, hemodialysis, resistance, stenotrophomonas maltophilia

## Abstract

Catheter-related bloodstream infections (CRBSIs) add to the morbidity and mortality of hemodialysis patients. *Stenotrophomonas maltophilia *is an extremely resistant, gram-negative, non-lactose-fermenting nosocomial bacterium that contributes significantly to mortality and morbidity. This bacterium is predominantly associated with community-acquired pneumonia, bacteremia, eye afflictions, biliary sepsis, urinary tract infection, skin and soft tissue infection, and very rarely chronic enteritis with colonic ulcers. Here, we present two cases that presented indolently and exhibit strikingly contrasting behaviors. The first case was a patient with primary hyperoxaluria on maintenance hemodialysis, who presented with CRBSI due to *S. maltophilia*, which responded appropriately to catheter removal and levofloxacin. The second patient, a case of diabetic nephropathy on maintenance hemodialysis, developed CRBSI due to *S. maltophilia*, which initially responded to catheter removal and levofloxacin but was later complicated dramatically by pneumonia and enteritis with colonic ulcers. These cases highlight the variable clinical presentation of this organism and emphasize the need for active surveillance in the dialysis unit.

## Introduction

*Stenotrophomonas maltophilia* is a multidrug-resistant, gram-negative, non-lactose-fermenting, and non-hemolytic nosocomial pathogen that is responsible for in-hospital mortality of 14%-69% and is associated predominantly with bacteremia, pneumonia, biliary sepsis, meningitis, urinary tract infection, dacryocystitis, and infection of the bones/joints [1]. Catheter-related bloodstream infection (CRBSI) due to *S. maltophilia* usually responds to timely catheter removal and appropriate antibiotic treatment [2]. The treatment algorithm for *S. maltophilia* is complicated due to poor susceptibility to existing antibiotic regimens and diverse mechanisms for acquired resistance to antibiotics [3]. Here, we describe two cases of CRBSI due to this nosocomial organism with variable clinical presentation and contrary outcomes.

## Case presentation

Case 1

A 38-year-old female patient with chronic kidney disease due to primary hyperoxaluria on maintenance hemodialysis twice weekly for seven years via a left tunneled internal jugular catheter presented with a low-grade fever for one week. She had a significant history of CRBSI due to different pathogens and multiple vascular access failures. On evaluation, she was febrile with a temperature of 101 degrees Fahrenheit and a respiratory rate of 24/min with normotension. Clinical examination revealed a few scattered crepitations in the right lower zone and decreased air entry in the right base of the lung. She had new-onset absence seizures during admission. Contrast-enhanced computerized tomography of the brain revealed normal study, and electroencephalogram revealed generalized spike-wave discharges of 2-4 Hz suggestive of metabolic etiology. She was started on levetiracetam (1,000 mg/day) for the seizures. Labs revealed hemoglobin (Hb) of 7.3 g/dL, white blood cell (WBC) count of 6,210/mm^3^, platelet of 169,000/mm^3^, normal liver function tests, creatinine of 6.9 mg/dL, urea of 57 mg/dL, and normal electrolytes (Table 1).

**Table 1:** Lab parameters of Case 1 on admission g/dL: grams/deciliter; mg/dL: milligrams/deciliter; meq/L: milliequivalents/liter; mm^3^: cubic millimeter; U/L: units/liter

Parameter	Values	Reference range
Hemoglobin (g/dL)	7.3	12-16
White blood cell count (/mm^3^)	6,210	4,500-11,000
Platelet count (/mm^3^)	169,000	150,000-370,000
Creatinine (mg/dL)	6.9	0.6-1.1
Urea (mg/dL)	57	15-40
Sodium (meq/L)	136	135-145
Potassium (meq/L)	4.9	3.5-5
Chloride (meq/L)	104	96-106
Bicarbonate (meq/L)	22	22-26
Total bilirubin (mg/dL)	0.4	0.2-1.2
Alanine transaminase (U/L)	22	19-40
Aspartate transaminase (U/L)	16	10-36

**Figure 1:**
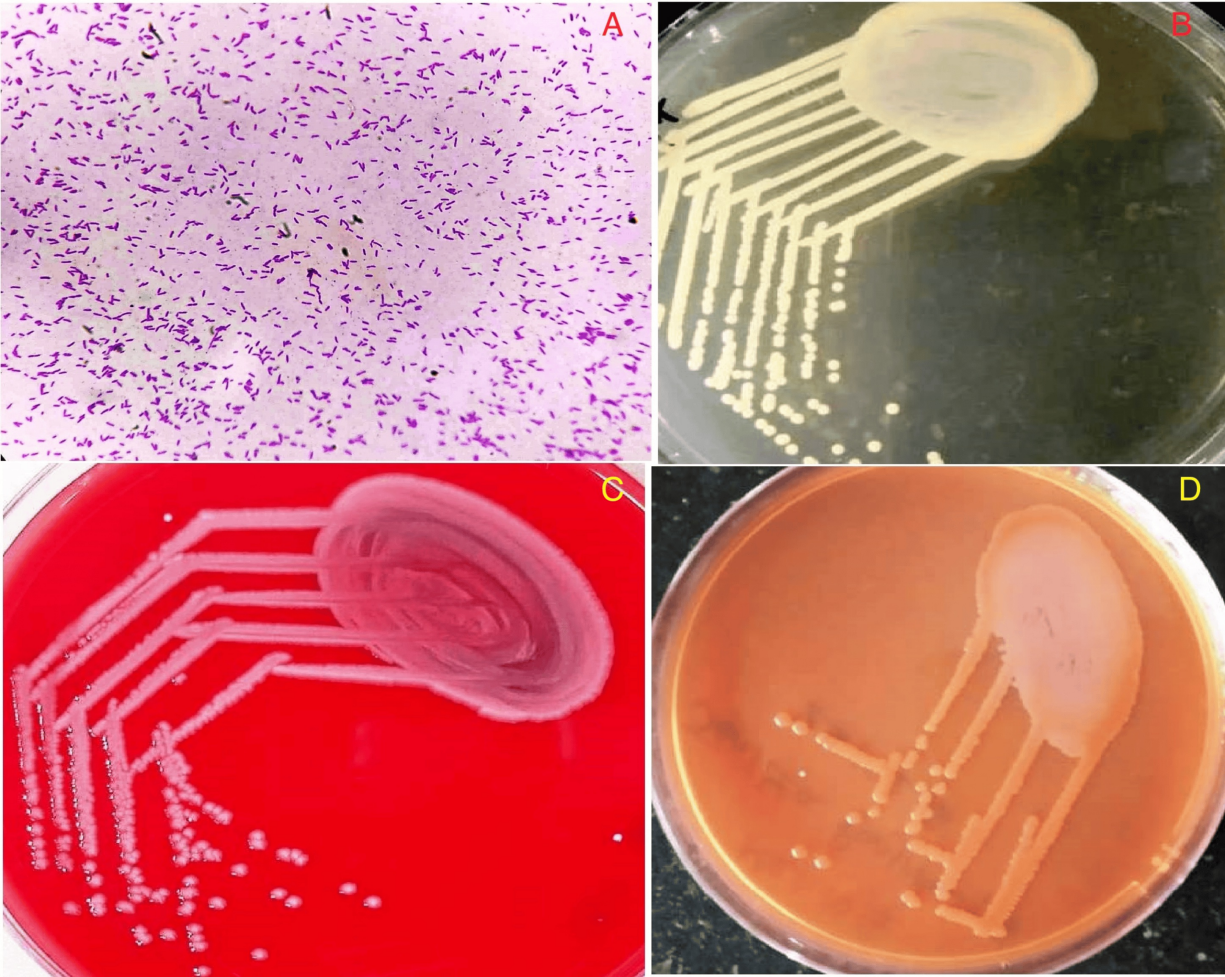
(A) Hematoxylin and eosin stain showing gram-negative, motile, polar flagellated bacterium suggestive of Stenotrophomonas maltophilia (magnification: 600 DPI; 400X). (B) Nutrient agar showing small circular, raised, yellowish pigmented colonies suggestive of Stenotrophomonas maltophilia (magnification: 600 DPI). (C) Blood agar classically showing non-hemolytic colonies of Stenotrophomonas maltophilia (magnification: 600 DPI). (D) MacConkey agar showing non-lactose-fermenting colonies of Stenotrophomonas maltophilia (magnification: 600 DPI) DPI: dots per inch

Chest X-ray revealed small right lower zone consolidation, and she was empirically started on injection of cefoperazone with sulbactam and azithromycin. However, the fever did not remit, and a blood culture drawn through a catheter revealed aerobic, gram-negative, motile, polar flagellated (Figure 1A) and catalase-positive organisms with small circular, raised, yellowish pigmented colonies on nutrient agar (Figure 1B) suggestive of *S. maltophilia*. A clinical decision was taken to remove the dialysis catheter with the insertion of a temporary left femoral dialysis catheter, and an injection of levofloxacin was initiated as per antibiotic susceptibility. The organism’s antibiotic susceptibility pattern demonstrated only sensitivity to levofloxacin with resistance to minocycline and cotrimoxazole. The patient had a good clinical response in terms of fever defervescence with three weeks of exposure to levofloxacin.

Case 2

A 65-year-old female patient with diabetic nephropathy on maintenance hemodialysis twice weekly in our unit for 1.5 years via a right internal jugular tunneled dialysis catheter presented with intermittent high-grade fever and nausea with decreased appetite for 10 days. On evaluation, she had a significant history of chills during hemodialysis sessions, and there was no systemic abnormality on clinical examination. Labs revealed Hb of 7.5 g/dL, WBC of 10,680/mm^3^, platelet of 231,000/mm^3^, creatinine of 6.6 mg/dL, urea of 62 mg/dL, normal serum electrolytes, and normal liver function tests (Table 2).

**Table 2:** Lab parameters of Case 2 on admission mg/dL: milligrams/deciliter; meq/L: milliequivalents/liter; mm^3^: cubic millimeter; U/L: units/liter

Parameter	Values	Reference range
Hemoglobin (g/dL)	7.5	12-16
White blood cell count (/mm^3^)	10,680	4,500-11,000
Platelet count (/mm^3^)	231,000	150,000-370,000
Creatinine (mg/dL)	6.6	0.6-1.1
Urea (mg/dL)	62	15-40
Sodium (meq/L)	135	135-145
Potassium (meq/L)	4.3	3.5-5
Chloride (meq/L)	106	96-106
Bicarbonate (meq/L)	21	22-26
Total bilirubin (mg/dL)	0.7	0.2-1.2
Alanine transaminase (U/L)	32	19-40
Aspartate transaminase (U/L)	24	10-36

The chest radiograph was normal. Blood cultures were drawn from the dialysis catheter, which revealed the growth of *S. maltophilia*. Blood agar classically showed non-hemolytic colonies of *S. maltophilia* (Figure 1C) and non-lactose fermentation on MacConkey agar (Figure 1D). The organism was susceptible to levofloxacin, and initiation of levofloxacin with the removal of the dialysis catheter resulted in an initial clinical response. Despite initial clinical improvement, she developed right-sided chest pain with desaturation on the sixth day of admission. She developed increasing desaturation, and clinical examination revealed harsh crepitations bilaterally with right-sided predominance. She developed spontaneous hemoptysis and hematochezia within two hours of ICU admission. Computerized tomography of the chest revealed patchy areas of consolidation with diffuse areas of atelectatic changes involving all segments of the right upper and lower lobes along with a few areas of consolidation in the apico-posterior segment of the left upper lobe. Repeat labs revealed Hb of 5 g/dL, WBC of 18,980/mm^3^, platelet of 195,000/mm^3^, prothrombin time of 13 seconds, international normalized ratio (INR) of 1.08, and activated partial thromboplastin time (aPTT) of 45 seconds (Table 3).

**Table 3:** Lab parameters of Case 2 on the sixth day after admission g/dL: grams/deciliter; mg/dL: milligrams/deciliter; meq/L: milliequivalents/liter; mm^3^: cubic millimeter; U/L: units/liter

Parameter	Values	Reference range
Hemoglobin (g/dL)	5	12-16
White blood cell count (/mm^3^)	18,980	4,500-11,000
Platelet count (/mm^3^)	195,000	150,000-370,000
Creatinine (mg/dL)	7.8	0.6-1.1
Urea (mg/dL)	102	15-40
Sodium (meq/L)	134	135-145
Potassium (meq/L)	5.1	3.5-5
Chloride (meq/L)	104	96-106
Bicarbonate (meq/L)	19	22-26
Total bilirubin (mg/dL)	0.6	0.2-1.2
Alanine transaminase (U/L)	38	19-40
Aspartate transaminase (U/L)	32	10-36

**Figure 2:**
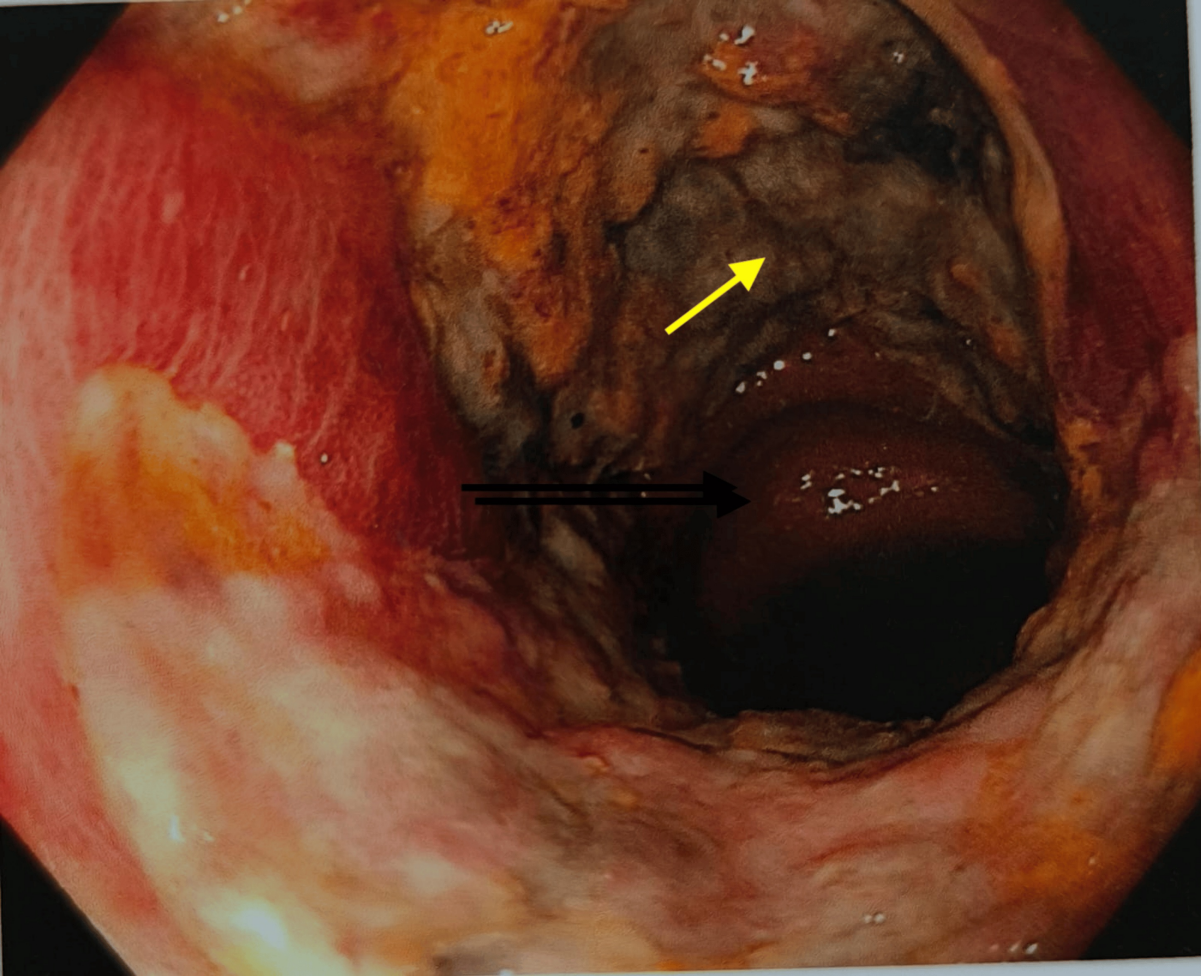
Colonoscopy showing a large circumferential friable ulcer (yellow arrow) with areas of necrosis and slough (magnification: 600 DPI) DPI: dots per inch

She was immediately dialyzed with optimal ultrafiltration along with packed red blood cell transfusion. Repeat blood cultures were sterile, and sputum culture was non-contributory. She was continued on levofloxacin along with the addition of meropenem. Colonoscopy for hematochezia was suggestive of a large circumferential friable ulcer with areas of necrosis and slough with ileitis (Figure 2).

Amoeba serology was negative. The histology of the ulcer showed only a necrotic ulcer with mild granulation tissue, which was non-contributory to the etiology. The fecal culture was negative. She was gradually weaned off from non-invasive ventilation with appropriate renal dosing of levofloxacin and meropenem. After three weeks of dual antibiotics, her respiratory and intestinal symptoms completely subsided, and she is continuing her thrice weekly hemodialysis via a new right femoral dialysis catheter.

## Discussion

*S. maltophilia* is a gram-negative, nosocomial, multidrug-resistant pathogen that has recently wreaked havoc on the health system with a wide affliction on all organ systems [1]. They have been retrieved from soil, wastewater, hemodialysis water distribution systems, dialysate samples, contaminated chlorhexidine antiseptics, and plastics, with a predilection to form biofilms [1,2]. A Greek multicenter study evaluating common gram-negative bacteria and antimicrobial resistance patterns in hemodialysis units found a prevalence of 13.5% for *S. maltophilia*, which is a testament to its presence and contamination of dialysate and water distribution systems in developing countries [4]. The source of infection in hemodialysis units due to this unusual organism is attributed to the contaminated hemodialysis water distribution system, contaminated dialysate water, O rings of reprocessed dialyzers, contaminated water for reprocessing dialyzers, adulterated chlorhexidine antiseptics, and contaminated handwashing agents [1,2,5].

*S. maltophilia*, previously known as *Pseudomonas maltophilia*, is an opportunistic pathogen that causes dacryocystitis, endophthalmitis, community-acquired pneumonia, biliary sepsis, meningitis, brain abscess, hepatic abscess, enterocolitis, arthritis, osteomyelitis, urinary tract infection, bacteremia, and implant/catheter infection [1,6]. The cause of pneumonia in our second case is due to this malevolent organism since there was no other documented culture positivity in our patient. The risk factors for mortality due to this organism include an elevated Charlson comorbidity index and sequential organ failure assessment (SOFA) score, septic shock, hypoalbuminemia, prior antibiotic use, quinolone resistance, need for invasive ventilation, presence of central venous or hemodialysis catheters, need for continuous renal replacement therapy, hematological malignancies, and chemotherapy [6]. *S. maltophilia* is a perfect example of a multi-drug-resistant bacteria, and this microbiological characteristic is conferred by the expression of multi-drug efflux pumps, alteration of bacterial lipopolysaccharide and target sites, acquisition of gene resistance by plasmid transfer, and antibiotic-inactivating enzymes [1,3]. The conventional antibiotics of choice for this organism include cotrimoxazole and fluoroquinolones [1,2,7]. However, in recent years, cotrimoxazole- and fluoroquinolone-resistant mutants have emerged due to the overexpression of SmeDEF or SmeVWX efflux pumps, which also confer resistance to second-line antibiotics, such as tigecycline [3]. The intrinsic resistance of this organism to all major conventional antibiotics is due to antibiotic-inactivating enzymes (L1/L2 β-lactamases and aminoglycoside-inactivating enzymes), chromosomally encoded "Qnr" pentapeptide repeat proteins, and the expression of multidrug efflux pumps, making it challenging in terms of treatment and outcome [7]. The higher rate of biofilm formation in this bacterium makes it unamenable to attack by the host immune system, chemical compounds, and susceptible antibiotics, which contribute to the increased clinical load of hemodialysis catheter-related bacteremia [2,8]. Traditionally, it is thought that *Stenotrophomonas* is intrinsically resistant to carbapenems; however, studies have shown that not all clinical isolates are resistant to carbapenems [9], which was proven by the fact that our second case had a good clinical response to meropenem.

Life-threatening colonic involvement is very rare in *S. maltophilia*, and it is due to the major extracellular protease (called StmPr1) produced by this bacterium, which is responsible for this severe hemorrhagic process and extensive tissue lesions in the form of colonic ulcer with ileitis [10]. Fecal carriage of this organism ranges between 10% and 30% [10], and this would have contributed to the negative fecal culture report in the background of susceptible antibiotics in our second case. However, by the principle of causality in infectious disease [11], we attribute that these colonic ulcers may have been caused by this unscrupulous organism considering the antecedent clinical events and treatment response. We could not ascertain the source of *S. maltophilia* contamination, and we postulate that some contamination might have occurred during the reprocessing of the dialyzer in the first case.

## Conclusions

Both cases highlight that clinical scenarios can dynamically vary in infections caused by *S. maltophilia* due to the acquisition of antimicrobial resistance, which is influenced by external environmental factors, leading to contrary clinical outcomes. These cases highlight the importance of active surveillance, refinement of standard hygiene practices, and universal precautions in hemodialysis units.
